# Coverage Analysis for High-Speed Railway Communications with Narrow-Strip-Shaped Cells over Suzuki Fading Channels

**DOI:** 10.3390/e26080657

**Published:** 2024-07-31

**Authors:** Shenghong Lin, Hongyan Wang, Weiyong Li, Jinyuan Wang

**Affiliations:** 1 Jiangsu Province Service Customization Network Application Engineering Research Center, Nanjing Vocational College of Information Technology, Nanjing 210023, China; wanghy@njcit.cn (H.W.); liwy@njcit.cn (W.L.); 2 School of Communications and Information Engineering, Nanjing University of Posts and Telecommunications, Nanjing 210003, China; jywang@njupt.edu.cn; 3 Chuan and Zang Smart Tourism Engineering Research Center of Colleges and Universities of Sichuan Province, Sichuan Tourism University, Chengdu 610100, China

**Keywords:** high-speed railway communications, coverage performance, narrow-strip-shaped cell, Suzuki fading, edge coverage probability, percentage of cell coverage area

## Abstract

Unlike circular cell coverage in public land mobile communications, narrow-strip-shaped cell coverage should be considered in high-speed railway (HSR) communications. Moreover, for the coverage analysis in HSR communications, most works ignore the effect of small-scale fading, which results in an inaccurate coverage performance evaluation. In this paper, we focus on the coverage analysis for HSR communications with narrow-strip-shaped cells over the Suzuki fading channel, where the composite channel fading includes path loss, lognormal shadowing, and Rayleigh-distributed small-scale fading. Based on the channel model, we first analyze the statistical characteristic of the received signal-to-noise ratio. Then, we derive analytical expressions of the edge coverage probability (ECP) and the percentage of cell coverage area (CCA). To link the edge coverage performance and the average coverage performance of a cell, we express the percentage of CCA as a summation of the ECP and a positive increment. As special cases, we also obtain the coverage performance expressions for the systems without small-scale fading. Through Monte Carlo simulations, the accuracy of the derived expressions is verified. Numerical results also show that the small-scale fading has a strong effect on coverage performance and cannot be ignored. In addition, the effects of key parameters are also discussed.

## 1. Introduction

As a comfortable and fast transportation tool, the high-speed railway (HSR) has been making great progress globally in recent decades. Thanks to its excellent performance, the HSR has improved people’s travel efficiency profoundly, expanding people’s living scope and promoting economic development. At present, China has the world’s largest HSR network, which is still expanding [1]. By the end of the medium–long-term railway network plan (2016–2030), China’s HSR network is expected to expand to around 200,000 km. To provide various mobile services for passengers on the high-speed train, advanced wireless communication techniques should also be explored.

For wireless communications, a good coverage scheme can significantly improve the system performance. To evaluate a certain coverage scheme, some coverage performance metrics, such as the percentage of cell coverage area (CCA), the edge coverage probability (ECP), and the edge outage probability, have been investigated. For traditional public land cellular communication systems with circular cells, the theoretical expression of the percentage of CCA was derived [2,3]. Moreover, the relationship between the percentage of CCA and the ECP was obtained [4], which links the edge coverage performance and the average coverage performance of a cell. Note that the overlap region between adjacent cells is not considered in [2,3,4]. To facilitate the handoff between different base stations (BSs), the percentage of CCA was further analyzed by considering the overlap region [5]. By considering the handoff, the relationship between the percentage of CCA and the ECP was also further analyzed [6]. However, due to the linear user distribution along the railway track, the circular cells used in [2,3,4,5,6] will lead to low coverage efficiency and radio resource waste in the coverage of HSR communication systems. Moreover, the coverage performance of HSR communication systems depends on the propagation environments (such as urban, suburban, rural, viaduct, cutting, station mountain, and river), which is different from that of conventional public land cellular communication (PLCC) systems.

Recently, some works have evaluated the coverage performance for HSR communication systems. To improve the coverage efficiency of circular cells, a coverage approach by decreasing the cell size for the HSR systems was proposed [7]. However, the coverage performance may be further improved by replacing circular cells with narrow-strip-shaped cells (also named linear cells, or elliptical cells). For a single-frequency-based HSR communication system in China, the narrow-strip-shaped coverage along the railway track was studied [8]. Moreover, the railway coverage in Spanish peninsular municipalities in 2018 and planned for 2024 was evaluated [9]. However, the specific performance indicators are not analyzed in [8,9]. By employing the modified Hata model [10] and the cooperative transmission scheme [11], the coverage probability, outage probability, transmission capacity, and number of BSs for HSR communication systems were evaluated. For HSR systems with narrow-strip-shaped cells, the percentage of CCA and the cell edge outage probability were analyzed [12]. For HSR systems with elliptical cells, a beamforming design problem was proposed to maximize the percentage of rail coverage [13]. However, only isolated cells were considered [12,13]. In practical systems, the handoff over the overlap region should be considered. To reduce handoff frequency, free-space optical communication frameworks were proposed for HSR communications [14,15]. By considering the handoff scheme, the percentage of CCA and the ECP were analyzed for HSR systems [16]. Moreover, the coverage analysis for HSR systems was also extended to the carrier aggregation scenario [17] and the cell-free massive multi-input multi-output scenario [18]. It should be emphasized that almost all the above works focus on large-scale fading and ignore small-scale fading. However, the small-scale fading has also a notable effect on coverage performance.

In this paper, we investigate the coverage performance of an HSR communication system. Unlike the circular cells used in PLCC systems [2,3,4,5,6], the narrow-strip-shaped cells are deployed along the railway track in this paper. Unlike previous studies that only consider the large-scale fading for HSR coverage [7,8,9,10,11,12,13,14,15,16,17,18], this paper considers the Suzuki fading channel, which includes both large-scale fading and small-scale fading. The main contributions of this paper are summarized as follows:

- We analyze the coverage performance at the cell edge. First, we analyze the statistical characteristics of the received signal-to-noise ratio (SNR). Then, according to the definition of ECP and the Gaussian–Hermite integral, we derive an analytical expression of the ECP. The ECP expression is shown to be a function of transmit power, cell radius, noise variance, standard deviation of shadow fading, HSR propagation environment, and SNR threshold.
- We analyze the average coverage performance of a cell, which can be characterized by the percentage of CCA. According to its mathematical definition, we derive its analytical expression. The percentage of CCA is also expressed as a function of system key parameters.
- We obtain a theoretical expression to link the ECP and the percentage of CCA. The percentage of CCA is expressed as the summation of the ECP and a positive increment. Thus, the relationship between the ECP and the percentage of CCA is established. As special cases, we also derive the theoretical expressions for the system without considering the small-scale fading.
- Some numerical results are provided. It is shown that the theoretical results match well with the simulation results, which verifies the accuracy of the derived theoretical expressions. Moreover, the small-scale fading has a strong effect on coverage performance, and thus it cannot be ignored. Furthermore, the effects of cell radius, transmit power, SNR threshold, propagation environment, and the shadow fading standard derivation on coverage performance are also provided.

The remainder of this paper is organized as follows. In Section 2, the system model of the HSR communication system is provided. Section 3 analyzes theoretical expressions of the ECP and percentage of CCA. Then, the relationship between the ECP and percentage of CCA is obtained in Section 4. Numerical results are presented in Section 5. Finally, conclusions are drawn in Section 6.

*Notation*: In this paper, italicized symbols denote scalar values; $\sqrt{\alpha }$ denotes the square root of a number α; $\log_{10}\left(\cdot \right)$ denotes the base-10 logarithm; ln(·) denotes the natural logarithm; exp(·) denotes the standard exponential function; |·| denotes the modulus of a complex number; $f_{X}\left(x\right)$ denotes the probability density function (PDF) of the variable *X*; $\mathrm{erfc}(x)=\frac{2}{\sqrt{\pi }}\int_{x}^{\infty }e^{-y^{2}}dy$ denotes the complementary error function; $\gamma \left(a,b\right)=\int_{0}^{b}t^{a-1}e^{-t}dt$ denotes the lower incomplete Gamma function; and $Q\left(x\right)=\int_{x}^{\infty }\frac{1}{\sqrt{2\pi }}\exp\left(\frac{t^{2}}{2}\right)dt$ denotes the Gaussian Q-function.

## 2. System Model

**Figure 1:**
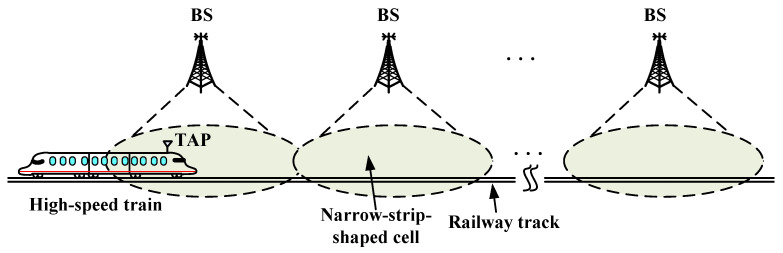
An HST communication system with narrow-strip-shaped cells.

As shown in Figure 1, we consider a classic HSR communication system in this paper. The system framework is applied to a high-speed train running on a straight railway track. The BSs are uniformly deployed along the railway track. To enlarge the coverage area, two directional antennas are installed on each BS, which point to each side of the BS. Thus, the whole system is covered by narrow-strip-shaped cells. Similar to [16,17,18], a train access point (TAP) is installed on the front roof of the train and taken as a mobile relay to deal with the rapid channel variation between the BS and the users in the train. As a result, the communication link between the BS and the users is divided into a backhaul link (i.e., the BS-TAP link) and an access link (i.e., the TAP-user link). The access link is similar to typical cellular communications, which has been widely studied in the literature. Therefore, the main challenge lies in the backhaul link. Without loss of generality, we concentrate on the backhaul link only in this paper.

Due to the deployment of BSs near the track, the vertical distance between each BS and the track can be ignored. Therefore, the horizontal distance between each BS and the TAP on the train can be considered the distance of these two nodes. We consider the downlink transmission from the BS to the TAP, and the cell radius is set to be *D*. When the horizontal distance between the BS and the TAP is *l*, the received signal *y* at the TAP can be written as [2]

(1) $$y=\sqrt{P_{T}}h\left(l\right)x+z,\;0\leq l\leq D$$

where $P_{T}$ is the transmit power of the BS; *x* is the transmit electrical signal with unit power; *z* is the additive white Gaussian noise with zero mean and variance $P_{N}$; and h(l) denotes the channel gain between the TAP and the BS.

In this paper, we consider a composite channel for HSR communications, which includes path loss, shadow fading, and small-scale fading. We assume that the encountered channel exhibits non-frequency-selective fading (i.e., flat-fading only). For a frequency-selective channel, orthogonal frequency division multiplexing schemes can be used to convert it into multiple flat-fading channels. Moreover, the effect of Doppler shift is ignored in the channel model because we can effectively eliminate it by employing some advanced compensation methods. Therefore, h(l) in (1) is given by

(2) $$h\left(l\right)=g\sqrt{PL(l)\cdot S}$$

where PL(l) is the path loss, *S* is the shadow fading, and *g* is the small-scale fading.

**Figure 2:**
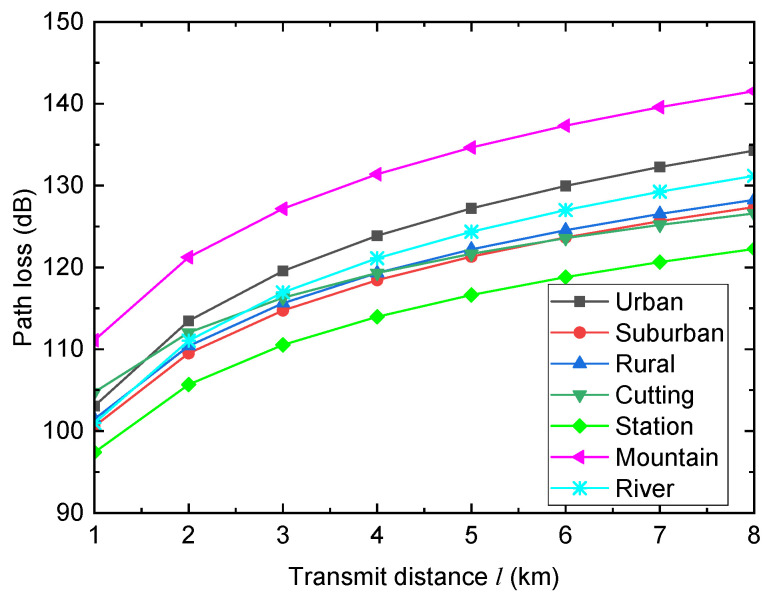
Path loss versus transmit distance under different HSR propagation environments when f=800 MHz, $h_{b}=20$ m, and $h_{T}=4$ m.

In (2), the path loss PL(l) for HSR communications can be modeled as [19]

(3) $$PL\left(l\right)=10^{-\frac{A+B\log_{10}\left(l\right)}{10}}$$

where *A* and *B* are defined as

(4) $$\left\{\begin{array}{c} A=\Delta_{1}+74.52+26.16\log_{10}\left(f\right)-13.82\log_{10}\left(h_{b}\right)-3.2{\left(\log_{10}\left(11.75h_{T}\right)\right)}^{2}\\ B=44.9-6.55\log_{10}\left(h_{b}\right)+\Delta_{2} \end{array}\right.$$

where *f* (in MHz) is the adopted frequency and $h_{b}$ (in m) and $h_{T}$ (in m) are effective antenna heights of the BS and the TAP. Moreover, $\Delta_{1}$ and $\Delta_{2}$ are the correction factors, which varies with the HSR propagation environment. Referring to [19], the values of $\Delta_{1}$ and $\Delta_{2}$ for various HSR propagation environments are provided in Table 1. Moreover, Figure 2 shows the path loss values (in dB) versus the transmit distance *l* (in km) under different HSR propagation environments when f=800 MHz, $h_{b}=20$ m, and $h_{T}=4$ m. As can be seen, propagation environments have strong effects on path loss performance. The larger the transmit distance is, the larger the path loss becomes. For each fixed transmit distance, the mountain environment always achieves the largest path loss, while the station environment always achieves the smallest path loss.

In (2), the shadow fading *S* in HSR communications follows a lognormal distribution. Thus, the PDF of *S* is given by

(5) $$f_{S}\left(s\right)=\frac{\xi }{\sqrt{2\pi }\sigma s}\exp\left(-\frac{{\left(10\log_{10}s\right)}^{2}}{2\sigma^{2}}\right),s>0$$

where ξ=10/ln10 and σ (in dB) is the standard deviation of $10\log_{10}S$.

In HSR communications, the small-scale fading follows the Rice distribution for viaduct scenarios over a plain area [20,21]. However, in mountain, hilly, or urban areas, or some other environment that is rich in scattering, Rayleigh fading is a reasonable model to capture the channel characteristics [22]. In this paper, the HSR scenario with rich scattering components is considered, and the small-scale fading *g* in (2) is modeled as the Rayleigh distribution [22]. As is known, the term $|g{|}^{2}$ follows an exponential distribution. Assuming that the mean value of $|g{|}^{2}$ is one, the PDF of $|g{|}^{2}$ is given by

(6) $$f_{|g{|}^{2}}\left(t\right)=\exp\left(-t\right),t\geq 0$$

According to the above analysis, the considered composite channel follows a Suzuki distribution (i.e., Rayleigh–lognormal distribution). The measurement results in [23] showed that the Suzuki distribution best describes the composite multipath/shadowing channels in HSR communications.

Moreover, the received SNR at the location *l* is expressed as

(7) $$\gamma \left(l\right)=\frac{P_{T}|h\left(l\right){|}^{2}}{P_{N}}=\Omega \left(l\right)|g{|}^{2}$$

where $\Omega \left(l\right)\overset{\Delta }{\;=}P_{T}\cdot PL\left(l\right)\cdot S/P_{N}$.

## 3. Coverage Performance Analysis

In this section, the statistical characteristics of the SNR are first analyzed. Then, the coverage performance (i.e., the ECP and the percentage of CCA) of the HSR communication system is analyzed.

### 3.1. Statistical Characteristics of SNR

Since *S* follows a lognormal distribution, Ω(l) also follows a lognormal distribution. Therefore, we can obtain the PDF of Ω(l) as

(8) $$\begin{array}{ccc} f_{\Omega (l)}\left(w\right) & = & \frac{P_{N}}{P_{T}\cdot PL\left(l\right)}f_{S}\left(\frac{P_{N}}{P_{T}\cdot PL\left(l\right)}w\right)\\ \; & = & \frac{\xi }{\sqrt{2\pi }\sigma w}\exp\left(-\frac{{\left(10\log_{10}\left(\frac{P_{N}w}{P_{T}\cdot PL\left(l\right)}\right)\right)}^{2}}{2\sigma^{2}}\right),w>0 \end{array}$$

According to (6) and (7), we can obtain the following conditional PDF as

(9) $$\begin{array}{ccc} f_{\gamma (l)|\Omega (l)}\left(r\left|w\right.\right) & = & \frac{1}{w}f_{|g{|}^{2}}\left(\frac{r}{w}\right)\\ \; & = & \frac{1}{w}\exp\left(-\frac{r}{w}\right),\;r\geq 0 \end{array}$$

From (8) and (9), we can obtain the PDF of the SNR as

(10) $$\begin{array}{ccc} f_{\gamma (l)}\left(r\right) & = & \int_{0}^{\infty }f_{\Omega (l)}\left(w\right)f_{\gamma (l)|\Omega (l)}\left(r\left|w\right.\right)dw\\ \; & = & \int_{0}^{\infty }\frac{\xi }{\sqrt{2\pi }\sigma w}\exp\left(-\frac{{\left(10\log_{10}\frac{P_{N}w}{P_{T}\cdot PL\left(l\right)}\right)}^{2}}{2\sigma^{2}}\right)\frac{1}{w}\exp\left(-\frac{r}{w}\right)dw \end{array}$$

Note that the SNR γ(l) follows a Gamma–lognormal distribution. It is challenging to obtain a closed-from expression of $f_{\gamma (l)}\left(r\right)$. To solve the integral in (10), we let $t=10\log_{10}\left(\frac{P_{N}w}{P_{T}\cdot PL\left(l\right)}\right)/\left(\sqrt{2}\sigma \right)$ and rewrite (10) as

(11) $$\begin{array}{ccc} f_{\gamma (l)}\left(r\right) & = & \int_{-\infty }^{\infty }\frac{\xi }{\sqrt{2\pi }\sigma }\exp\left(-t^{2}\right)\frac{\exp\left(-\frac{r}{\frac{P_{T}\cdot PL\left(l\right)}{P_{N}}10^{\frac{\sqrt{2}\sigma t}{10}}}\right)}{{\left(\frac{P_{T}\cdot PL\left(l\right)}{P_{N}}10^{\frac{\sqrt{2}\sigma t}{10}}\right)}^{2}}\frac{P_{T}\cdot PL\left(l\right)}{P_{N}}10^{\frac{\sqrt{2}\sigma t}{10}}\frac{\ln10}{10}\sqrt{2}\sigma dt\\ \; & = & \frac{P_{N}}{\sqrt{\pi }P_{T}\cdot PL\left(l\right)}\int_{-\infty }^{\infty }\exp\left(-t^{2}\right)10^{-\frac{\sqrt{2}\sigma t}{10}}\exp\left(-\frac{P_{N}r}{P_{T}\cdot PL\left(l\right)}10^{-\frac{\sqrt{2}\sigma t}{10}}\right)dt \end{array}$$

According to [24], the Gauss–Hermite integral is given by

(12) $$\int_{-\infty }^{\infty }e^{-x^{2}}f\left(x\right)dx≅\sum_{i=1}^{N_{p}}H_{i}f\left(x_{i}\right)$$

where $t_{i}$ and $H_{i}$ are the base point and weight factor of the $N_{p}$-order Gauss–Hermite approximation.

Solving (11) by using (12), we have

(13) $$f_{\gamma (l)}\left(r\right)≅\frac{P_{N}}{\sqrt{\pi }P_{T}\cdot PL\left(l\right)}\sum_{i=1}^{N_{p}}H_{i}10^{-\frac{\sqrt{2}\sigma t_{i}}{10}}\exp\left(-\frac{P_{N}r}{P_{T}\cdot PL\left(l\right)}10^{-\frac{\sqrt{2}\sigma t_{i}}{10}}\right)$$

**Remark** **1.** *If the small-scale fading is not considered, the Suzuki channel (i.e., path loss + shadow fading + Rayleigh fading) becomes the lognormal shadowing channel (i.e., path loss + shadow fading). In this case, the SNR γ(l) in (7) reduces to $\gamma^{\prime }\left(l\right)=\Omega \left(l\right)$. Therefore, we can conclude that, if small-scale fading is ignored, we will overestimate or underestimate the quality of the received signal, resulting in inaccurate coverage performance analysis. Moreover, in this case, the PDF of SNR (13) reduces to $f_{\gamma^{\prime }\left(l\right)}\left(r\right)=f_{\Omega (l)}\left(r\right)$.*

### 3.2. ECP Analysis

In this subsection, the ECP is analyzed. As is known, the ECP $P_{e}$ is defined as the probability that the SNR at the cell edge (i.e., l=D) exceeds a given threshold $\gamma_{\mathrm{th}}$. Therefore, we have

(14) $$\begin{array}{ccc} P_{e} & = & \mathrm{Pr}\left\{\gamma \left(D\right)\geq \gamma_{\mathrm{th}}\right\}\\ \; & = & \int_{\gamma_{\mathrm{th}}}^{\infty }f_{\gamma (D)}\left(r\right)dr \end{array}$$

By solving (14), we obtain the following theorem.

**Theorem** **1.** *For the considered HSR communication system over the Suzuki fading channels, the ECP is given by*

(15) $$P_{e}=\frac{1}{\sqrt{\pi }}\sum_{i=1}^{N_{p}}H_{i}\exp\left(-\frac{P_{N}\gamma_{\mathrm{th}}}{P_{T}\cdot PL\left(D\right)}10^{-\frac{\sqrt{2}\sigma t_{i}}{10}}\right)$$

**Proof.**  See Appendix A. □

**Corollary** **1.** *If the small-scale fading is not considered, the Suzuki channel becomes the lognormal shadowing channel. In this case, the ECP in Theorem 1 reduces to*

(16) $$\begin{array}{ccc} P_{e}^{\prime } & = & \int_{\gamma_{\mathrm{th}}}^{\infty }\frac{\xi }{\sqrt{2\pi }\sigma w}\exp\left(-\frac{{\left(10\log_{10}\frac{P_{N}w}{P_{T}\cdot PL\left(D\right)}\right)}^{2}}{2\sigma^{2}}\right)dw\\ \; & = & \frac{1}{2}\mathrm{erfc}\left(\frac{10\log_{10}\left(\frac{P_{N}\gamma_{\mathrm{th}}}{P_{T}\cdot PL\left(D\right)}\right)}{\sqrt{2}\sigma }\right) \end{array}$$

### 3.3. Percentage of CCA Analysis

The percentage of CCA is another important indicator for coverage analysis. For the HSR communication with narrow-strip-shaped cells, the percentage of CCA is defined as

(17) $$\begin{array}{ccc} P_{a} & = & \frac{1}{D}\int_{0}^{D}\mathrm{Pr}\left\{\gamma \left(l\right)\geq \gamma_{\mathrm{th}}\right\}dl\\ \; & = & \frac{1}{D}\int_{0}^{D}\int_{\gamma_{\mathrm{th}}}^{\infty }f_{\gamma (l)}\left(r\right)drdl \end{array}$$

By solving (17), we obtain the following theorem.

**Theorem** **2.** *For the considered HSR communication system over the Suzuki fading channels, the percentage of CCA is given by*

(18) $$P_{a}=\frac{1}{\sqrt{\pi }D}\frac{10}{B}\sum_{i=1}^{N_{p}}H_{i}{\left(\frac{P_{T}}{P_{N}\gamma_{\mathrm{th}}}10^{\frac{\sqrt{2}\sigma t_{i}-A}{10}}\right)}^{\frac{10}{B}}\gamma \left(\frac{10}{B},\frac{P_{N}\gamma_{\mathrm{th}}}{P_{T}}10^{\frac{A-\sqrt{2}\sigma t_{i}}{10}D^{\frac{B}{10}}}\right)$$

*where γ(·,·) is the lower incomplete Gamma function.*

**Proof.**  See Appendix B. □

**Corollary** **2.** *If the small-scale fading is not considered, the Suzuki channel becomes the lognormal shadowing channel. In this case, the percentage of CCA in Theorem 2 reduces to*

(19) $$\begin{array}{ccc} P_{a}^{\prime } & = & \frac{1}{2D}10^{\frac{10\log_{10}\left(\frac{P_{T}}{P_{N}\gamma_{\mathrm{th}}}\right)-A}{B}}\left[e^{\frac{\ln10}{B}\cdot 10\log_{10}\left(\frac{P_{N}\gamma_{\mathrm{th}}}{P_{T}\cdot 10^{\frac{A+B\log_{10}\left(D\right)}{10}}}\right)}\mathrm{erfc}\left(\frac{10\log_{10}\left(\frac{P_{N}\gamma_{\mathrm{th}}}{P_{T}\cdot 10^{\frac{A+B\log_{10}\left(D\right)}{10}}}\right)}{\sqrt{2}\sigma }\right)\right.\\ \; & + & \left.e^{\frac{\sigma^{2}{\left(\ln10\right)}^{2}}{2B^{2}}}\mathrm{erfc}\left(\frac{\sigma \ln10}{\sqrt{2}B}-\frac{10\log_{10}\left(\frac{P_{N}\gamma_{\mathrm{th}}}{P_{T}\cdot 10^{\frac{A+B\log_{10}\left(D\right)}{10}}}\right)}{\sqrt{2}\sigma }\right)\right] \end{array}$$

**Proof.**  See Appendix C. □

## 4. Relationship between ECP and Percentage of CCA

In Section 3, the derived ECP and percentage of CCA are isolated expressions, but the relationship between them is not provided. In this section, we try to link the ECP and the percentage of CCA to show their relationship.

According to the definition of the percentage of CCA, we can further obtain

(20) $$\begin{array}{ccc} P_{a} & = & \frac{1}{D}\int_{0}^{D}\left[\int_{\gamma_{\mathrm{th}}}^{\infty }f_{\gamma (l)}\left(r\right)dr\right]dl\\ \; & = & \frac{1}{D}\int_{0}^{D}\left[1-\int_{0}^{\gamma_{\mathrm{th}}}f_{\gamma (l)}\left(r\right)dr\right]dl\\ \; & = & 1-\frac{1}{D}\left[{\left.l\int_{0}^{\gamma_{\mathrm{th}}}f_{\gamma (l)}\left(r\right)dr\right|}_{0}^{D}-\int_{0}^{D}l\int_{0}^{\gamma_{\mathrm{th}}}\frac{\partial }{\partial l}f_{\gamma (l)}\left(r\right)drdl\right]\\ \; & = & P_{e}+\frac{1}{D}\int_{0}^{D}l\int_{0}^{\gamma_{\mathrm{th}}}\frac{\partial }{\partial l}f_{\gamma (l)}\left(r\right)drdl \end{array}$$

By solving (20), we obtain the following theorem.

**Theorem** **3.** *For the considered HSR communication system under the Suzuki fading channels, the relationship between the percentage of CCA $P_{a}$ and the ECP $P_{e}$ is given by*

(21) $$\begin{array}{ccc} P_{a} & = & P_{e}+\frac{1}{\sqrt{\pi }D}\sum_{i=1}^{N_{p}}H_{i}{\left(10^{\frac{\sqrt{2}\sigma t_{i}-A}{10}}\frac{P_{T}}{P_{N}\gamma_{\mathrm{th}}}\right)}^{\frac{10}{B}}\gamma \left(\frac{10}{B}+1,\frac{P_{N}\gamma_{\mathrm{th}}}{P_{T}}10^{\frac{A-\sqrt{2}\sigma t_{i}}{10}}D^{\frac{B}{10}}\right) \end{array}$$

**Proof.**  See Appendix D. □

**Remark** **2.** *Theorem 3 links the ECP and the percentage of CCA. As can be seen, the percentage of CCA is equal to the ECP plus a positive increment. As is known, the worst coverage performance is achieved at the cell edge, and thus the average coverage performance of the cell is better than the coverage performance at the cell edge.*

**Corollary** **3.** *If the small-scale fading is not considered, the Suzuki channel becomes the lognormal shadowing channel. In this case, the relationship between the percentage of CCA $P_{a}^{\prime }$ and the ECP $P_{e}^{\prime }$ becomes*

(22) $$\begin{array}{ccc} P_{a}^{\prime } & = & P_{e}^{\prime }+\frac{1}{D}{\left(\frac{P_{N}\gamma_{\mathrm{th}}}{P_{T}}\right)}^{-\frac{10}{B}}10^{-\frac{A}{B}}\exp\left(\frac{\sigma^{2}{(\ln10)}^{2}}{2B^{2}}\right)\\ \; & \times & \left[1-Q\left(\frac{10\log_{10}\left(\frac{P_{N}\gamma_{\mathrm{th}}}{P_{T}\cdot PL\left(D\right)}\right)}{\sigma }-\frac{\sigma \ln10}{B}\right)\right] \end{array}$$

**Proof.**  See Appendix E. □

## 5. Numerical Results

In this section, some classic numerical results are shown to validate the cell coverage analysis for HSR communications. Monte Carlo simulation results are also given to verify the correctness of the derived expressions. In the simulation figures of this section, the simulation (or theoretical) results with *g* correspond to the Suzuki channel, while the simulation (or theoretical) results without *g* correspond to the lognormal shadowing channel. The main simulation parameters are listed in Table 2.

### 5.1. Results of ECP

**Figure 3:**
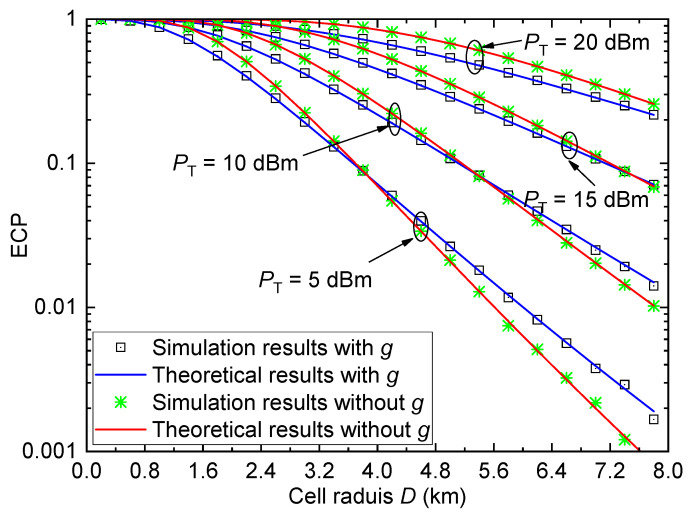
ECP versus cell radius for the urban environment with different $P_{T}$ when $\gamma_{\mathrm{th}}=1$ and σ=6.

**Figure 4:**
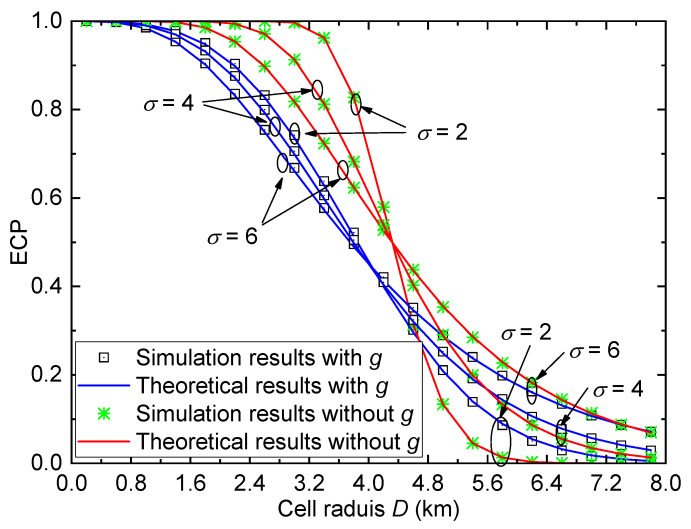
ECP versus cell radius for the urban environment with different σ when $\gamma_{\mathrm{th}}=1$, and $P_{T}=15\;\mathrm{dBm}$.

**Figure 5:**
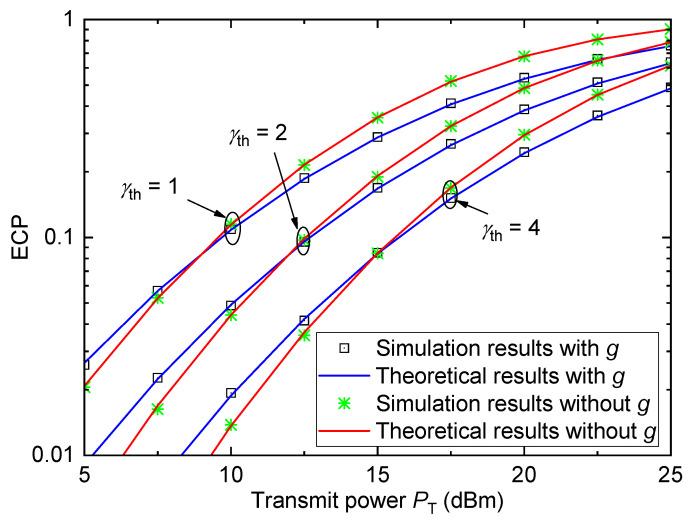
ECP versus transmit power for the urban environment with different $\gamma_{\mathrm{th}}$ when σ=6 and D=5km.

**Figure 6:**
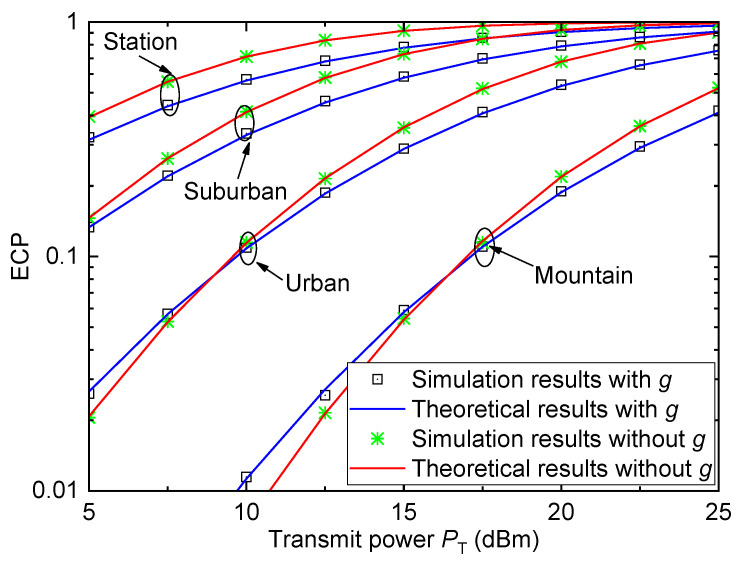
ECP versus transmit power for different HSR propagation environments when $\gamma_{\mathrm{th}}=1$, σ=6, and D=5km.

In this subsection, we provide some results of ECP in Figure 3, Figure 4, Figure 5 and Figure 6. To facilitate the comparison, the coverage performance for the system without considering small-scale fading *g* is also provided.

Figure 3 shows the ECP $P_{e}$ versus the cell radius *D* for the urban environment with different transmit power values $P_{T}$ when $\gamma_{\mathrm{th}}=1$ and σ=6. It can be observed that the ECP value reduces with *D*, which indicates that the larger coverage region will result in a lower coverage probability at the cell edge. For a large transmit power $P_{T}$, a large SNR can be obtained, and thus a better coverage performance can be achieved. Moreover, when the cell radius *D* is small, the ECP of the system with the small-scale fading is smaller than that of the system without the small-scale fading. However, when *D* is large, the ECP of the system with the small-scale fading is larger than that of the system without the small-scale fading. This indicates that the ECP performance is overestimated at small *D* and underestimated at large *D* when the small-scale fading is not considered, resulting in an inaccurate performance evaluation. Therefore, the small-scale fading has a strong effect on ECP performance and cannot be ignored in practical coverage analysis.

Figure 4 plots the ECP $P_{e}$ versus the cell radius *D* for the urban environment with different shadow fading standard derivations σ when $\gamma_{\mathrm{th}}=1$ and $P_{T}=15\;\mathrm{dBm}$. For a small cell radius *D*, the ECP reduces with the increase in standard derivation σ. However, for a large cell radius *D*, the ECP increases with σ. Similar to Figure 3, the ECP performance of the system with *g* is worse than that of the system without *g* when *D* is small, and the performance of the system with *g* is better than that of the system without *g* when *D* is large. Therefore, the small-scale fading cannot be ignored.

Figure 5 provides the relationship between the ECP $P_{e}$ and the transmit power $P_{T}$ for the urban environment with different SNR thresholds $\gamma_{\mathrm{th}}$ when σ=6 and D=5km. As can be seen, the ECP values increase with the increase in $P_{T}$, which is the same as that in Figure 3. With the increase in $\gamma_{\mathrm{th}}$, the ECP values reduce. This is obvious because a larger SNR threshold will result in a worse coverage performance. Moreover, the ECP performance of VLC with small-scale fading is better than that without small-scale fading for small values of $P_{T}$; however, the observation is opposite for large values of $P_{T}$.

Figure 6 shows the ECP $P_{e}$ versus the transmit power $P_{T}$ for different HSR propagation environments when $\gamma_{\mathrm{th}}=1$, σ=6, and D=5km. As can be observed, the coverage performance varies with the HSR propagation environment. The station environment achieves the best ECP performance, the suburban environment achieves the second best ECP performance, the urban environment achieves the second worst ECP performance, and the mountain environment achieves the worst ECP performance. These conclusions are obvious by observing the path loss values of different HSR propagation environments in Figure 2.

For Figure 3, Figure 4, Figure 5 and Figure 6, all theoretical results match well with the simulation results, which verify the accuracy of the derived ECP expression.

### 5.2. Results of Percentage of CCA

**Figure 7:**
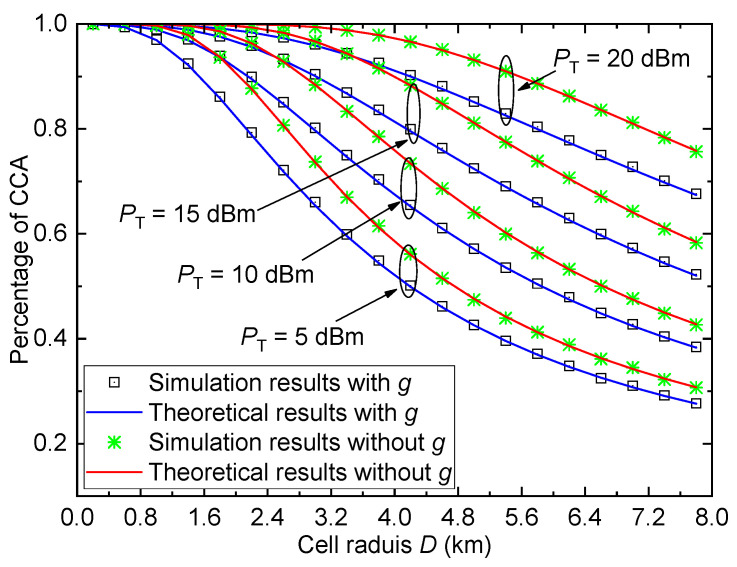
Percentage of CCA versus cell radius for the urban environment with different $P_{T}$ when $\gamma_{\mathrm{th}}=1$, and σ=6.

**Figure 8:**
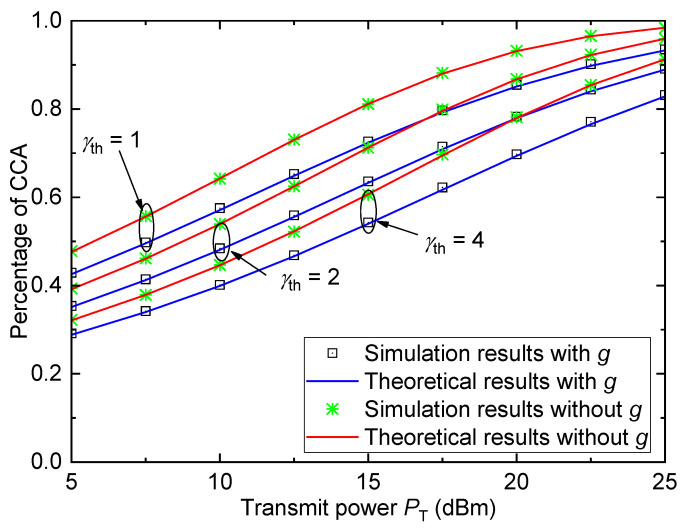
Percentage of CCA versus transmit power for the urban environment with different $\gamma_{\mathrm{th}}$ when σ=6 and D=5km.

**Figure 9:**
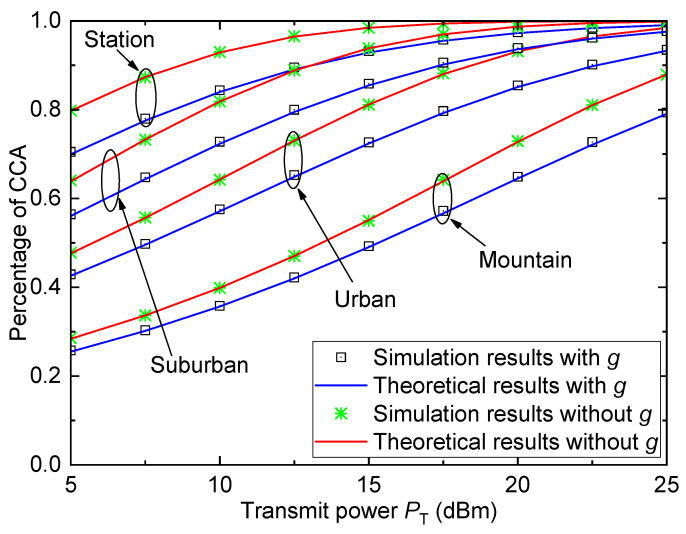
Percentage of CCA versus transmit power for different HSR propagation environments when $\gamma_{\mathrm{th}}=1$, σ=6, and D=5km.

**Figure 10:**
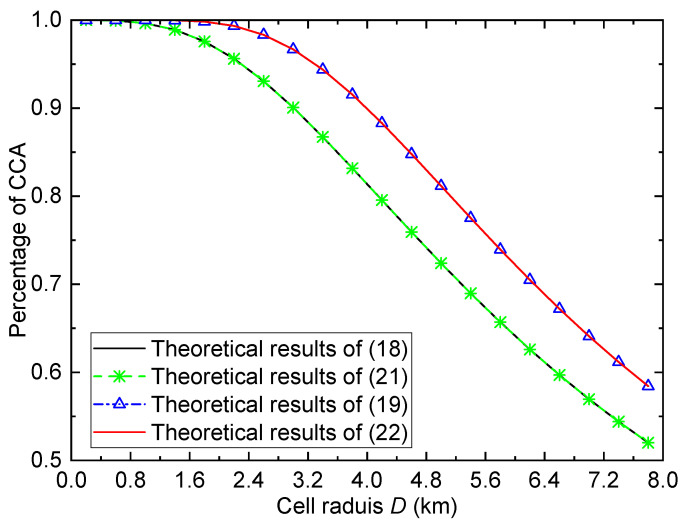
Different results of the percentage of CCA versus cell radius for the urban environment when $\gamma_{\mathrm{th}}=1$, σ=6, and $P_{T}=15\;\mathrm{dBm}$.

In this subsection, we provide some results of the percentage of CCA in Figure 7, Figure 8, Figure 9 and Figure 10. To facilitate the comparison, the coverage performance for the system without considering small-scale fading is also provided.

Figure 7 shows the percentage of CCA $P_{a}$ versus the cell radius *D* for the urban environment with different transmit power values $P_{T}$ when $\gamma_{\mathrm{th}}=1$ and σ=6. Obviously, the values of the percentage of CCA reduce with the increase in *D*, which is similar to the conclusion in Figure 3. Moreover, compared with Figure 3, we can observe that for fixed $P_{T}$ and *D*, the ECP values are always smaller than the percentage of CCA values, which indicates that the average coverage performance of the cell outperforms the cell edge coverage performance. Furthermore, when *D* changes from 0 to 8 km, the performance of the system without considering small-scale fading is always better than that of the system with the small-scale fading. Therefore, the average coverage performance will be overestimated if the small-scale fading is ignored.

Figure 8 shows the percentage of CCA $P_{a}$ versus the transmit power $P_{T}$ for the urban environment with different SNR thresholds $\gamma_{\mathrm{th}}$ when σ=6 and D=5km. Similar to Figure 5, the percentage of CCA also increases with the increase in transmit power $P_{T}$ or the decrease in SNR threshold $\gamma_{\mathrm{th}}$. Moreover, the percentage of CCA in this figure is larger than the ECP in Figure 5 for fixed $P_{T}$ and $\gamma_{\mathrm{th}}$. As can be seen, the performance of the system with small-scale fading is worse than that without considering the small-scale fading. These conclusions are similar to that in Figure 7.

Figure 9 shows the percentage of CCA $P_{e}$ versus the transmit power $P_{T}$ for different HSR propagation environments when $\gamma_{\mathrm{th}}=1$, σ=6, and D=5km. As can be seen, the HSR propagation environments have strong effects on coverage performance. For different HSR environments, the average coverage performance from best to worst is ranked as station > suburban > urban > mountain, which is the same as that in Figure 6. We can also observe that the average coverage performance is overestimated if the small-scale fading is not considered.

For Figure 7, Figure 8 and Figure 9, the gaps between all theoretical results and simulation results are sufficiently small and can be ignored, which verify the accuracy of the derived percentage of CCA expression.

Moreover, as stated in the previous section, Equations (21) and (22) provide the relationships between the ECP and the percentage of CCA. To verify the accuracy of the derived expressions ((21) and (22)), Figure 10 shows different results of the percentage of CCA versus the cell radius for the urban environment when $\gamma_{\mathrm{th}}=1$, σ=6, and $P_{T}=15\;\mathrm{dBm}$. To facilitate the comparison, Equations (18) and (19) are also provided, which can be used to evaluate the percentage of CCA directly. However, Equations (21) and (22) are the functions of $P_{e}$ and $P_{e}^{\prime }$, respectively. The value of $P_{e}$ in (21) is determined by (15), while the value of $P_{e}^{\prime }$ in (22) is determined by (16). From the figure, it can be observed that the theoretical results of (21) match well with those of (18), and the theoretical results of (22) match well with those of (19). These results verify the accuracy of the derived expressions (21) and (22).

## 6. Conclusions

This paper investigates the coverage performance for HSR communications with narrow-strip-shaped cells. Unlike previous works, the small-scale fading is considered in the channel model, and thus a composite channel named the Suzuki fading channel is considered. The main conclusions are summarized as follows:

- For the HSR system with small-scale fading, analytical expressions of the ECP and the percentage of CCA are derived, respectively. To facilitate the comparison, the coverage performance indicator expressions for the system without considering the small-scale fading are also derived. Numerical results verify the accuracy of the derived expressions.
- To link the edge coverage performance and the average coverage performance of the whole cell, we derive the relationship between the ECP and the percentage of CCA. Specifically, the percentage of CCA is expressed as the summation of the ECP and a positive increment. Therefore, the average coverage performance of a cell is always better than the edge coverage performance.
- The HSR propagation environments have strong effects on coverage performance. For example, the station scenario has a smaller path loss and thus has a better coverage performance, while the mountain scenario has a larger path loss and thus has a worse coverage performance.
- It is shown that the ECP or the percentage of CCA will be overestimated or underestimated if the small-scale fading is not considered. This indicates that, for the coverage analysis in HSR communications, the small-scale fading cannot be ignored.
- The cell radius, transmit power, SNR threshold, and shadow fading standard derivation also have strong effects on coverage performance. Specifically, the coverage performance improves with the decrease in cell radius, the increase in transmit power, the decrease in SNR threshold, or the decrease in shadow fading standard derivation.

## Figures and Tables

**Table 1:** Values of correction factors for different HSR propagation environments.

Scenarios	$\Delta_{1}$	$\Delta_{2}$
Urban	−20.47	−1.82
Suburban	$5.74\log_{10}\left(h_{b}\right)-30.42$	−6.72
Rural	$6.43\log_{10}\left(h_{b}\right)-30.44$	−6.71
Cutting	−18.78	$51.34\log_{10}\left(h_{b}\right)-78.99$
Station	$34.29\log_{10}\left(h_{b}\right)-70.75$	−8.86
Mountain	$-66.26\log_{10}\left(h_{b}\right)+73.77$	$-84.14\log_{10}\left(h_{b}\right)+106.79$
River	$8.79\log_{10}\left(h_{b}\right)-33.99$	−2.93

**Table 2:** Main simulation parameters.

Parameters	Symbols	Values
Carrier frequency	*f*	800 MHz
BS antenna height	$h_{b}$	20 m
TAP antenna height	$h_{T}$	4 m
Noise variance	$P_{N}$	$10^{-14}$
Order of Gauss–Hermite approximation	$N_{p}$	40

## Data Availability

The data presented in this study are available on request from the corresponding author.

## Appendix A. Proof of Theorem 1

Substituting (13) into (14), we have

(A1) $$P_{e}=\int_{\gamma_{\mathrm{th}}}^{\infty }\frac{P_{N}}{\sqrt{\pi }P_{T}\cdot PL\left(D\right)}\sum_{i=1}^{N_{p}}H_{i}10^{-\frac{\sqrt{2}\sigma t_{i}}{10}}\exp\left(-\frac{P_{N}r}{P_{T}\cdot PL\left(D\right)}10^{-\frac{\sqrt{2}\sigma t_{i}}{10}}\right)dr$$

Change the order of integral and summation in (A1), we can further express $P_{e}$ as

(A2) $$P_{e}=\frac{P_{N}}{\sqrt{\pi }P_{T}\cdot PL\left(D\right)}\sum_{i=1}^{N_{p}}H_{i}10^{-\frac{\sqrt{2}\sigma t_{i}}{10}}\int_{\gamma_{\mathrm{th}}}^{\infty }\exp\left(-\frac{P_{N}r}{P_{T}\cdot PL\left(D\right)}10^{-\frac{\sqrt{2}\sigma t_{i}}{10}}\right)dr$$

Then, let $m=\frac{P_{N}r}{P_{T}\cdot PL\left(D\right)}10^{-\frac{\sqrt{2}\sigma t_{i}}{10}}$, Equation (A2) can be written as

(A3) $$\begin{array}{ccc} P_{e} & = & \frac{P_{N}}{\sqrt{\pi }P_{T}\cdot PL\left(D\right)}\sum_{i=1}^{N_{p}}H_{i}10^{-\frac{\sqrt{2}\sigma t_{i}}{10}}\int_{\frac{P_{N}\gamma_{\mathrm{th}}}{P_{T}\cdot PL\left(D\right)}10^{-\frac{\sqrt{2}\sigma t_{i}}{10}}}^{\infty }\exp\left(-m\right)\frac{P_{T}\cdot PL\left(D\right)}{P_{N}}10^{\frac{\sqrt{2}\sigma t_{i}}{10}}dm\\ \; & = & \frac{1}{\sqrt{\pi }}\sum_{i=1}^{N_{p}}H_{i}\exp\left(-\frac{P_{N}\gamma_{\mathrm{th}}}{P_{T}\cdot PL\left(D\right)}10^{-\frac{\sqrt{2}\sigma t_{i}}{10}}\right) \end{array}$$

Therefore, Equation (15) holds.

## Appendix B. Proof of Theorem 2

According to (14) and (15), we can obtain

(A4) $$\int_{\gamma_{\mathrm{th}}}^{\infty }f_{\gamma (l)}\left(r\right)dr=\frac{1}{\sqrt{\pi }}\sum_{i=1}^{N_{p}}H_{i}\exp\left(-\frac{P_{N}\gamma_{\mathrm{th}}}{P_{T}\cdot PL\left(l\right)}10^{-\frac{\sqrt{2}\sigma t_{i}}{10}}\right)$$

Then, substituting (A4) into (17), we have

(A5) $$P_{a}=\frac{1}{D}\int_{0}^{D}\frac{1}{\sqrt{\pi }}\sum_{i=1}^{N_{p}}H_{i}\exp\left(-\frac{P_{N}\gamma_{\mathrm{th}}}{P_{T}\cdot PL\left(l\right)}10^{-\frac{\sqrt{2}\sigma t_{i}}{10}}\right)dl$$

Changing the order of integral and summation, we can further obtain

(A6) $$\begin{array}{c} P_{a}=\frac{1}{\sqrt{\pi }D}\sum_{i=1}^{N_{p}}H_{i}\int_{0}^{D}\exp\left(-\frac{P_{N}\gamma_{\mathrm{th}}10^{\frac{A-\sqrt{2}\sigma t_{i}}{10}}}{P_{T}}l^{\frac{B}{10}}\right)dl \end{array}$$

Let $m=\frac{P_{N}\gamma_{\mathrm{th}}10^{\frac{A-\sqrt{2}\sigma t_{i}}{10}}}{P_{T}}l^{\frac{B}{10}}$, Equation (A6) can be further written as

(A7) $$\begin{array}{ccc} P_{a} & = & \frac{1}{\sqrt{\pi }D}\sum_{i=1}^{N_{p}}H_{i}\int_{0}^{\frac{P_{N}\gamma_{\mathrm{th}}10^{\frac{A-\sqrt{2}\sigma t_{i}}{10}}}{P_{T}}D^{\frac{B}{10}}}e^{-m}\frac{10}{B}{\left(\frac{P_{T}\cdot 10^{\frac{\sqrt{2}\sigma t_{i}-A}{10}}}{P_{N}\gamma_{\mathrm{th}}}m\right)}^{\frac{10}{B}-1}\frac{P_{T}}{P_{N}\gamma_{\mathrm{th}}}10^{\frac{\sqrt{2}\sigma t_{i}-A}{10}}dm\\ \; & = & \frac{1}{\sqrt{\pi }D}\frac{10}{B}\sum_{i=1}^{N_{p}}H_{i}{\left(\frac{P_{T}}{P_{N}\gamma_{\mathrm{th}}}10^{\frac{\sqrt{2}\sigma t_{i}-A}{10}}\right)}^{\frac{10}{B}}\int_{0}^{\frac{P_{N}\gamma_{\mathrm{th}}10^{\frac{A+B\log_{10}\left(D\right)-\sqrt{2}\sigma t_{i}}{10}}}{P_{T}}}e^{-m}\cdot m^{\frac{10}{B}-1}dm \end{array}$$

Moreover, the lower incomplete Gamma function is defined as [24], Equation (6.5.2).

(A8) $$\gamma \left(s,x\right)=\int_{0}^{x}t^{s-1}e^{-t}dt$$

By applying (A8) for (A7), we can obtain (18).

## Appendix C. Proof of Corollary 2

When the small-scale fading is not considered, substituting $f_{\gamma^{\prime }\left(l\right)}\left(r\right)=f_{\Omega (l)}\left(r\right)$ into (17) and using Corollary 1, we obtain the percentage of CCA as

(A9) $$\begin{array}{ccc} P_{a}^{\prime } & = & \frac{1}{D}\int_{0}^{D}\int_{\gamma_{\mathrm{th}}}^{\infty }\frac{\xi }{\sqrt{2\pi }\sigma r}\exp\left(-\frac{{\left(10\log_{10}\left(\frac{P_{N}r}{P_{T}\cdot PL\left(l\right)}\right)\right)}^{2}}{2\sigma^{2}}\right)drdl\\ \; & = & \frac{1}{D}\int_{0}^{D}\frac{1}{2}\mathrm{erfc}\left(\frac{10\log_{10}\left(\frac{P_{N}\gamma_{\mathrm{th}}}{P_{T}\cdot PL\left(l\right)}\right)}{\sqrt{2}\sigma }\right)dl \end{array}$$

Then, substituting (3) into (A9) and letting $t=10\log_{10}\left(\frac{P_{N}\gamma_{\mathrm{th}}}{P_{T}\cdot 10^{-\frac{A+B\log_{10}\left(l\right)}{10}}}\right)/\left(\sqrt{2}\sigma \right)$, we have

(A10) $$\begin{array}{ccc} P_{a}^{\prime } & = & \frac{\sigma \ln10}{\sqrt{2}DB}10^{\frac{10\log_{10}\left(\frac{P_{T}}{P_{N}\gamma_{\mathrm{th}}}\right)-A}{B}}\int_{-\infty }^{\frac{10\log_{10}\left(\frac{P_{N}\gamma_{\mathrm{th}}}{P_{T}\cdot 10^{\frac{A+B\log_{10}\left(D\right)}{10}}}\right)}{\sqrt{2}\sigma }}\mathrm{erfc}(t)10^{\frac{\sqrt{2}\sigma t}{B}}dt\\ \; & = & \frac{\sigma \ln10}{\sqrt{2}DB}10^{\frac{10\log_{10}\left(\frac{P_{T}}{P_{N}\gamma_{\mathrm{th}}}\right)-A}{B}}\int_{-\infty }^{\frac{10\log_{10}\left(\frac{P_{N}\gamma_{\mathrm{th}}}{P_{T}\cdot 10^{\frac{A+B\log_{10}\left(D\right)}{10}}}\right)}{\sqrt{2}\sigma }}\mathrm{erfc}\left(t\right)e^{\frac{\sqrt{2}\sigma \ln10}{B}t}dt \end{array}$$

According to [25], we have the following identity as

(A11) $$\int e^{bt}\mathrm{erfc}\left(at\right)dt=\frac{1}{b}\left[e^{bt}\mathrm{erfc}\left(at\right)-e^{\frac{b^{2}}{4a^{2}}}\mathrm{erf}\left(\frac{b}{2a}-at\right)\right]$$

By using (A11), we have

(A12) $$\begin{array}{ccc} P_{a}^{\prime } & = & \frac{1}{2D}10^{\frac{10\log_{10}\left(\frac{P_{T}}{P_{N}\gamma_{\mathrm{th}}}\right)-A}{B}}\left[e^{\frac{\ln10}{B}\cdot 10\log_{10}\left(\frac{P_{N}\gamma_{\mathrm{th}}}{P_{T}\cdot 10^{\frac{A+B\log_{10}\left(D\right)}{10}}}\right)}\mathrm{erfc}\left(\frac{10\log_{10}\left(\frac{P_{N}\gamma_{\mathrm{th}}}{P_{T}\cdot 10^{\frac{A+B\log_{10}\left(D\right)}{10}}}\right)}{\sqrt{2}\sigma }\right)\right.\\ \; & - & \left.e^{\frac{\sigma^{2}{\left(\ln10\right)}^{2}}{2B^{2}}}\mathrm{erf}\left(\frac{\sigma \ln10}{\sqrt{2}B}-\frac{10\log_{10}\left(\frac{P_{N}\gamma_{\mathrm{th}}}{P_{T}\cdot 10^{\frac{A+B\log_{10}\left(D\right)}{10}}}\right)}{\sqrt{2}\sigma }\right)+e^{\frac{\sigma^{2}{\left(\ln10\right)}^{2}}{2B^{2}}}\right] \end{array}$$

Then, using erfc(x)=1−erf(x), we can obtain (19).

## Appendix D. Proof of Theorem 3

According to (13) and (3), $\partial f_{\gamma (l)}\left(r\right)/\partial l$ can be written as

(A13) $$\begin{array}{ccc} \frac{\partial f_{\gamma (l)}\left(r\right)}{\partial l} & = & \frac{\partial }{\partial l}\left[\frac{P_{N}}{\sqrt{\pi }P_{T}}\sum_{i=1}^{N_{p}}H_{i}10^{\frac{A+B\log_{10}\left(l\right)-\sqrt{2}\sigma t_{i}}{10}}\exp\left(-\frac{P_{N}r}{P_{T}}10^{\frac{A+B\log_{10}\left(l\right)-\sqrt{2}\sigma t_{i}}{10}}\right)\right]\\ \; & = & \frac{P_{N}}{\sqrt{\pi }P_{T}}\sum_{i=1}^{N_{p}}H_{i}10^{\frac{A-\sqrt{2}\sigma t_{i}}{10}}\cdot \frac{\partial }{\partial l}\left[l^{\frac{B}{10}}\exp\left(-\frac{P_{N}r}{P_{T}}10^{\frac{A-\sqrt{2}\sigma t_{i}}{10}}l^{\frac{B}{10}}\right)\right]\\ \; & = & \frac{P_{N}B}{10\sqrt{\pi }P_{T}}\sum_{i=1}^{N_{p}}H_{i}10^{\frac{A-\sqrt{2}\sigma t_{i}}{10}}\left(l^{\frac{B}{10}-1}-\frac{P_{N}r}{P_{T}}10^{\frac{A-\sqrt{2}\sigma t_{i}}{10}}l^{\frac{B}{5}-1}\right)e^{-\frac{P_{N}r}{P_{T}}10^{\frac{A-\sqrt{2}\sigma t_{i}}{10}}l^{\frac{B}{10}}} \end{array}$$

Then, we have

(A14) $$\begin{array}{ccc} \; & \; & \int_{0}^{D}l\int_{0}^{\gamma_{\mathrm{th}}}\frac{\partial }{\partial l}f_{\gamma (l)}\left(r\right)drdl=\frac{P_{N}B}{10\sqrt{\pi }P_{T}}\sum_{i=1}^{N_{p}}H_{i}10^{\frac{A-\sqrt{2}\sigma t_{i}}{10}}\\ \; & \; & \times \int_{0}^{D}l\underset{\overset{\Delta }{\;=}I_{1}}{\underset{︸}{\int_{0}^{\gamma_{\mathrm{th}}}\left(l^{\frac{B}{10}-1}-\frac{P_{N}}{P_{T}}10^{\frac{A-\sqrt{2}\sigma t_{i}}{10}}l^{\frac{B}{5}-1}r\right)\exp\left(-\frac{P_{N}}{P_{T}}10^{\frac{A-\sqrt{2}\sigma t_{i}}{10}}l^{\frac{B}{10}}r\right)dr}}dl \end{array}$$

For the integral term $I_{1}$ in (A14), we let $m=\frac{P_{N}}{P_{T}}10^{\frac{A-\sqrt{2}\sigma t_{i}}{10}}l^{\frac{B}{10}}r$ and obtain

(A15) $$\begin{array}{ccc} I_{1} & = & \frac{P_{T}}{P_{N}}10^{\frac{\sqrt{2}\sigma t_{i}-A}{10}}l^{-1}\int_{0}^{\frac{P_{N}}{P_{T}}10^{\frac{A-\sqrt{2}\sigma t_{i}}{10}}l^{\frac{B}{10}}\gamma_{\mathrm{th}}}\left(1-m\right)\exp\left(-m\right)dm\\ \; & = & l^{\frac{B}{10}-1}\gamma_{\mathrm{th}}\exp\left(-\frac{P_{N}}{P_{T}}10^{\frac{A-\sqrt{2}\sigma t_{i}}{10}}l^{\frac{B}{10}}\gamma_{\mathrm{th}}\right) \end{array}$$

Substituting (A15) into (A14), we have

(A16) $$\begin{array}{ccc} \int_{0}^{D}l\int_{\gamma_{\mathrm{th}}}^{\infty }\frac{\partial }{\partial l}f_{\gamma (l)}\left(r\right)drdl & = & \frac{BP_{N}\gamma_{\mathrm{th}}}{10\sqrt{\pi }P_{T}}\sum_{i=1}^{N_{p}}H_{i}10^{\frac{A-\sqrt{2}\sigma t_{i}}{10}}\\ \; & \times & \underset{\overset{\Delta }{\;=}I_{2}}{\underset{︸}{\int_{0}^{D}l^{\frac{B}{10}}\exp\left(-\frac{P_{N}}{P_{T}}10^{\frac{A-\sqrt{2}\sigma t_{i}}{10}}l^{\frac{B}{10}}\gamma_{\mathrm{th}}\right)dl}} \end{array}$$

For the integral term $I_{2}$ in (A16), we let $m=\frac{P_{N}}{P_{T}}10^{\frac{A-\sqrt{2}\sigma t_{i}}{10}}l^{\frac{B}{10}}\gamma_{\mathrm{th}}$ and obtain

(A17) $$\begin{array}{ccc} I_{2} & = & \int_{0}^{\frac{P_{N}}{P_{T}}10^{\frac{A-\sqrt{2}\sigma t_{i}}{10}}D^{\frac{B}{10}}\gamma_{\mathrm{th}}}\left(10^{\frac{\sqrt{2}\sigma t_{i}-A}{10}}\frac{P_{T}m}{P_{N}\gamma_{\mathrm{th}}}\right)\exp\left(-m\right){\left(10^{\frac{\sqrt{2}\sigma t_{i}-A}{10}}\frac{P_{T}}{P_{N}\gamma_{\mathrm{th}}}\right)}^{\frac{10}{B}}\frac{10}{B}m^{\frac{10}{B}-1}dm\\ \; & = & \frac{10}{B}{\left(10^{\frac{\sqrt{2}\sigma t_{i}-A}{10}}\frac{P_{T}}{P_{N}\gamma_{\mathrm{th}}}\right)}^{\frac{10}{B}+1}\int_{0}^{\frac{P_{N}}{P_{T}}10^{\frac{A-\sqrt{2}\sigma t_{i}}{10}}D^{\frac{B}{10}}\gamma_{\mathrm{th}}}m^{\frac{10}{B}}\exp\left(-m\right)dm\\ \; & = & \frac{10}{B}{\left(10^{\frac{\sqrt{2}\sigma t_{i}-A}{10}}\frac{P_{T}}{P_{N}\gamma_{\mathrm{th}}}\right)}^{\frac{10}{B}+1}\gamma \left(\frac{10}{B}+1,\frac{P_{N}}{P_{T}}10^{\frac{A-\sqrt{2}\sigma t_{i}}{10}}D^{\frac{B}{10}}\gamma_{\mathrm{th}}\right) \end{array}$$

Substituting (A16) and (A17) into (20), we can obtain (21).

## Appendix E. Proof of Corollary 3

Similar to (20), we can obtain the relationship between the ECP and the percentage of CCA as

(A18) $$P_{a}^{\prime }=P_{e}^{\prime }+\frac{1}{D}\int_{0}^{D}l\int_{0}^{\gamma_{\mathrm{th}}}\frac{\partial }{\partial l}f_{\gamma^{\prime }\left(l\right)}\left(r\right)drdl$$

In this case, $\partial f_{\gamma^{\prime }\left(l\right)}\left(r\right)/\partial l$ can be written as

(A19) $$\begin{array}{ccc} \frac{\partial f_{\gamma^{\prime }\left(l\right)}\left(r\right)}{\partial l} & = & \frac{\partial f_{\Omega (l)}\left(r\right)}{\partial l}\\ \; & = & \frac{\xi }{\sqrt{2\pi }\sigma r}\frac{\partial }{\partial l}\exp\left(-\frac{{\left(10\log_{10}\left(\frac{P_{N}r}{P_{T}\cdot PL\left(l\right)}\right)\right)}^{2}}{2\sigma^{2}}\right)\\ \; & = & -\frac{B\xi^{2}\log_{10}\left(\frac{P_{N}r}{P_{T}\cdot PL\left(l\right)}\right)}{\sigma^{3}l\sqrt{2\pi }r}\cdot \exp\left(-\frac{{\left(10\log_{10}\left(\frac{P_{N}r}{P_{T}\cdot PL\left(l\right)}\right)\right)}^{2}}{2\sigma^{2}}\right) \end{array}$$

Then, we have

(A20) $$\begin{array}{ccc} \int_{0}^{\gamma_{\mathrm{th}}}\frac{\partial }{\partial l}f_{\gamma^{\prime }\left(l\right)}\left(r\right)dr & = & -\frac{B\xi^{2}}{\sigma^{3}l\sqrt{2\pi }}\int_{0}^{\gamma_{\mathrm{th}}}\frac{\log_{10}\left(\frac{P_{N}r}{P_{T}\cdot PL\left(l\right)}\right)}{r}\cdot \exp\left(-\frac{{\left(10\log_{10}\left(\frac{P_{N}r}{P_{T}\cdot PL\left(l\right)}\right)\right)}^{2}}{2\sigma^{2}}\right)dr\\ \; & = & \frac{B}{\sigma l\sqrt{2\pi }\ln10}\exp\left(-{\frac{\left(10\log_{10}\left(\frac{P_{N}\gamma_{\mathrm{th}}}{P_{T}\cdot PL\left(l\right)}\right)\right)}{2\sigma^{2}}}^{2}\right) \end{array}$$

Furthermore, let $m=10\log_{10}\left(\frac{P_{N}\gamma_{\mathrm{th}}}{P_{T}}10^{\frac{A+B\log_{10}\left(l\right)}{10}}\right)/\sigma$, we have

(A21) $$\begin{array}{ccc} \int_{0}^{D}l\int_{0}^{\gamma_{\mathrm{th}}}\frac{\partial }{\partial l}f_{\gamma^{\prime }\left(l\right)}\left(r\right)drdl & = & \frac{B}{\sigma \sqrt{2\pi }\ln10}\int_{0}^{D}\exp\left(-{\frac{\left(10\log_{10}\left(\frac{P_{N}\gamma_{\mathrm{th}}}{P_{T}}10^{\frac{A+B\log_{10}\left(l\right)}{10}}\right)\right)}{2\sigma^{2}}}^{2}\right)dl\\ \; & = & \frac{1}{\sqrt{2\pi }}{\left(\frac{P_{N}\gamma_{\mathrm{th}}}{P_{T}}\right)}^{-\frac{10}{B}}10^{-\frac{A}{B}}\int_{-\infty }^{\frac{10\log_{10}\left(\frac{P_{N}\gamma_{\mathrm{th}}}{P_{T}}10^{\frac{A+B\log_{10}\left(D\right)}{10}}\right)}{\sigma }}e^{-\frac{m^{2}}{2}}10^{\frac{\sigma m}{B}}dm \end{array}$$

Then, we use $a^{x}=e^{\ln a^{x}}=e^{x\ln a}$, and obtain

(A22) $$\begin{array}{ccc} \; & \; & \int_{0}^{D}l\int_{0}^{\gamma_{\mathrm{th}}}\frac{\partial }{\partial l}f_{\gamma^{\prime }\left(l\right)}\left(r\right)drdl=\frac{1}{\sqrt{2\pi }}{\left(\frac{P_{N}\gamma_{\mathrm{th}}}{P_{T}}\right)}^{-\frac{10}{B}}10^{-\frac{A}{B}}\int_{-\infty }^{\frac{10\log_{10}\left(\frac{P_{N}\gamma_{\mathrm{th}}}{P_{T}}10^{\frac{A+B\log_{10}\left(D\right)}{10}}\right)}{\sigma }}e^{-\frac{m^{2}}{2}+\frac{\sigma \ln10}{B}m}dm\\ \; & \; & ={\left(\frac{P_{N}\gamma_{\mathrm{th}}}{P_{T}}\right)}^{-\frac{10}{B}}10^{-\frac{A}{B}}\exp\left(\frac{\sigma^{2}{(\ln10)}^{2}}{2B^{2}}\right)\left[1-Q\left(\frac{10\log_{10}\left(\frac{P_{N}\gamma_{\mathrm{th}}}{P_{T}\cdot PL\left(D\right)}\right)}{\sigma }-\frac{\sigma \ln10}{B}\right)\right] \end{array}$$

Substituting (A22) into (A18), we can obtain (22).
